# The Dark Side of Leisure Time: Analysis of the Predictive Effects Between Boredom, Internet Usage Habits, and Gambling Behaviors

**DOI:** 10.3390/brainsci15060598

**Published:** 2025-06-03

**Authors:** Esra Emir, Elif Akça, Adela Badau, Dana Badau

**Affiliations:** 1 Sport Sciences Faculty, İstanbul Aydin University, İstanbul 34158, Turkey; emiresraa@gmail.com (E.E.); elifakca@aydin.edu.tr (E.A.); 2 Faculty of Physical Education and Mountain Sports, Transilvania University of Brasov, 500036 Brasov, Romania; dana.badau@unitbv.ro

**Keywords:** boredom, internet usage habits, night-time gambling behaviors, leisure time, adults

## Abstract

**Background/Objectives:** This study aimed to identify the relationships between individuals’ perceptions of leisure boredom, internet usage habits, and gambling motivations, focusing on analyzing the predictive effects by age category in adults. **Methods**: The study employs quantitative research methods, adopting a relational survey model. The sample group comprises 310 adult (94 female and 214 male) individuals aged 18 and over. Data were collected using the Leisure Boredom Scale (LBS), targeting two subscales: negative (Boredom) and positive (Satisfaction) attitudes toward leisure time, the Leisure Internet Use Scale (LIUS) targeting four forms of leisure, and the Gambling Motivation Scale (GMS) with the following subscales: socialization, entertainment/excitement, escapism, and money-making. In the analysis conducted with SPSS 25.0, independent samples t-test, ANOVA, correlation, and regression analyses were applied. **Results**: The findings indicate that leisure boredom significantly predicts internet usage and gambling motivations. Notably, individuals who spend their leisure time on passive activities were found to have higher rates of internet usage, habits, digital addiction, and gambling behavior. The positive relationship between internet usage habits, digital addictions, and risky behaviors such as gambling becomes particularly pronounced among young adults. **Conclusions:** Leisure time management is critical for individuals’ psychological well-being. Cognitive awareness programs, digital literacy education, and promoting participation in healthy leisure activities are recommended as key strategies to prevent risky behaviors.

## 1. Introduction

Leisure time is a period when individuals can rest, engage in recreation, or dedicate time to personal interests, independent of work and obligations [1]. It significantly affects personal satisfaction, healthy living, social relationships, and overall quality of life [2]. Leisure time is not merely a time segment; it is also seen as an opportunity for self-actualization [3]. In this regard, properly utilizing leisure time is crucial for individual psychological well-being and public health. However, it has been observed that leisure time may not be equally satisfying for all individuals, and how this time is perceived and utilized is shaped by personal perspectives. In this context, individuals may experience negative emotions such as boredom during leisure time and feelings of inefficiency, emptiness, or monotony [4]. The perception of boredom becomes more prominent when individuals cannot find adequate activities or stimuli to engage with during their leisure time [5]. This situation can have psychological and social effects, as boredom is associated with low motivation, depression, and anxiety [5]. Furthermore, the perception of boredom is not only an individual experience but can also be influenced by societal and cultural factors. The way societies and individuals perceive the concept of leisure time is an important variable affecting this perception [6]. 

The negative utilization of leisure time can lead to psychological, physical, and social problems. One such negative outcome is the misuse of leisure time, which is associated with the perception of boredom [7,8]. When individuals experience boredom during their leisure time, they often turn to harmful and addictive activities as distractions. This process can lead to dependencies, such as internet addiction, which is one of the greatest dangers of the modern age. Individuals may spend long hours on online platforms to escape boredom and monotony, reducing physical activity and potentially resulting in social isolation [9,10]. Similarly, gambling addiction can arise as a consequence of boredom, with individuals engaging in risky and harmful behaviors. Gambling can serve as a means for those seeking excitement to make their leisure time feel more valuable and to escape the monotony [11]. Such addictions not only jeopardize individuals’ psychological and physical health but also weaken their social relationships and negatively impact their overall quality of life [12]. Therefore, the practical and healthy use of leisure time is essential in preventing the adverse consequences of boredom perception.

The perception of boredom emerges from individuals’ personal preferences and environmental factors. In today’s fast-paced world, people are constantly exposed to digital stimuli and tend to seek more stimulation and speed, which can sometimes lead to feelings of boredom [4]. Consequently, the relationship between leisure time and boredom directly correlates with the digitalization of modern society and its rapidly evolving social norms. The connection between leisure time and internet use has become an essential social reality for contemporary youth and adults. The internet provides various activities, including entertainment content, social media platforms, video watching, online gaming, and information gathering. In this context, internet usage is one of the most common and easily accessible ways to spend leisure time [13]. As such, the internet significantly influences how individuals engage in leisure activities, shaping their experiences within a social and cultural framework. Boredom is another major factor that drives individuals to use the internet. The time spent online often increases to alleviate feelings of boredom [13,14,15]. As people invest more time in online activities to fill their leisure time, this behavior can gradually become a habit. Digital platforms, particularly social media and online games, help relieve feelings of boredom and emptiness. However, excessive involvement in these activities may lead to psychological issues such as social isolation and depression [5]. Moreover, excessive internet use can result in neglect of physical activities, face-to-face social interactions, and creative hobbies [16,17,18,19]. In this regard, the internet’s role in leisure activities can generate positive and negative outcomes.

Beyond boredom during leisure time and internet use, the reasons why individuals engage in gambling also become an essential issue. Gambling often starts as a source of entertainment and excitement, but can develop into a behavior that leads to addiction over time [20]. Gambling is closely linked to the perception of boredom, as it offers an activity through which individuals can fulfill their desire for risk-taking, pursue quick rewards, and experience instant gratification. In this context, boredom and internet usage may provoke riskier behaviors, such as gambling [21,22]. The widespread use of the internet has also increased the popularity of online gambling. Online gambling has led to a rise in gambling addiction in the digital realm, making gambling behaviors more accessible and anonymous [23]. Online gambling platforms allow individuals to gamble with the desire to quickly win or lose large sums of money, which increases the risk of addiction. While gambling may be perceived as an exciting and rewarding activity in the short term, it can lead to psychological, financial, and social issues in the long run [24]. Excessive gambling can harm individuals’ social relationships and result in financial ruin [25].

Significant psychological and social factors influence how individuals spend their leisure time. While boredom can trigger internet use, the digital entertainment and anonymity provided by the internet may encourage individuals to engage in gambling [26]. The relationship among these three factors is influenced by motivations such as fulfilling psychological needs, seeking excitement, and obtaining rewards. However, excessive engagement in these behaviors can adversely affect individuals’ psychological well-being. Boredom during leisure time, unconscious and excessive internet use, and potential addiction issues related to gambling represent significant social and psychological challenges faced in modern society. A deeper examination of these phenomena can facilitate the development of effective interventions that encourage individuals to spend their leisure time more healthily and productively, such as engaging in physical activity, participating in social or cultural events, or focusing on personal development. This study aims to identify perceptions of boredom during leisure time, patterns of internet usage, and reasons for gambling among adults across various socio-economic variables, and to analyze the association effects by exploring the relationships between these variables.

## 2. Materials and Methods

### 2.1. Participants

In total, 310 adults, including 96 women and 214 men, aged 18 and over, were selected through purposive sampling and engaged in online gambling, betting, or gaming to participate in the study. The average age of the participants is 40.18 ± 14.167. The inclusion criteria for the study are being over 18 years old and having participated in at least one form of chance-based gambling. Participation in the survey was voluntary and adhered to the principles outlined in the Declaration of Helsinki and approved by the Ethics Committee of the Faculty of Physical Education and Mountain Sports, Transilvania University of Brasov, No. 68/04.03.2025. 

The minimum required sample size was calculated using G*Power 3.1.9.7 software. Based on an effect size of 0.15, a 1% margin of error, and a statistical power of 95%, the required sample size was determined to be 143 participants. The study’s participant group consisted of adult individuals residing in Istanbul. An online survey (Google Forms) was used as the data collection tool. This approach was adopted to facilitate access to individuals with specific characteristics, who constitute the study’s target population, and to examine the relevant behaviors in depth. Although this method offers the advantage of rapid access to the target audience, it carries a risk of sampling bias due to the absence of random selection. This limitation may affect the generalizability of the findings to the broader adult population.

### 2.2. Study Design and Procedures

This study employed quantitative research techniques, specifically descriptive and relational survey models. After selecting the participants and obtaining informed consent, they received the questionnaires and instructions for completing them on the Google Forms platform for data collection. The online form included information about the participants’ age and gender in the initial section.

### 2.3. Assessment Tools

The study involved administering three questionnaires to subjects who had engaged in at least one form of chance-based gambling.

1. The Leisure Boredom Scale (LBS), a standardized questionnaire developed by Iso-Ahola and Weissinger (1990) [27], measures individual differences in boredom perception during leisure time. The validity and reliability study of the Turkish version for adult adaptation was conducted by Kara, Gürbüz, and Öncü (2014) [28]. The Turkish version used in this study consists of 10 items targeting two subdimensions, each containing 5 items. The “Boredom” subdimension reflects an individual’s negative attitude toward leisure activities (e.g., “I usually don’t enjoy what I do in my leisure time, but I don’t know what else to do”). Conversely, the “Satisfaction” subdimension showcases an individual’s positive perspective on leisure time (e.g., “The idea of leisure time excites me” or “Leisure time energizes me”). A 5-point Likert scale was employed to evaluate responses, ranging from (1) “strongly disagree” to (5) “strongly agree”, with (3) representing “neither agree nor disagree.” The Cronbach’s alpha coefficient for internal consistency of the scale in this study was calculated to be 0.74.
2. The Leisure Internet Usage Scale (LIUS), developed by Şimşek and Çevik [29], was employed in this study. The dimensions of the LIUS were designed by considering leisure activities viewed as enjoyable forms of internet use (e.g., information and personal development, shopping, multimedia sources, and social interaction). It includes items that cover the factors influencing these usage patterns. The scale is a 5-point Likert-type scale, where (1) means “strongly disagree” and (5) means “strongly agree”, with (3) indicating “neither agree nor disagree”, and consists of 4 dimensions with a total of 16 items. In this study, the Cronbach’s alpha coefficient for the internal consistency of the scale was calculated to be 0.72.
3. The Gambling Motives Scale, developed by Lee et al. (2007) [30], was utilized to understand participants’ motivations for gambling. This scale employs a 5-point Likert-type format ranging from (1) “strongly disagree” to (5) “strongly agree”, with (3) indicating “neither agree nor disagree.” It assesses four subdimensions of gambling motivation: socialization, entertainment/excitement, escapism, and money-making. The scale comprises a total of 35 items, and scores in each subdimension reflect individuals’ attitudes toward that specific motivation. The Turkish adaptation of the scale was conducted by Arcan and Karancı [31]. The Cronbach’s alpha coefficient for internal consistency of the scale in this study was calculated to be 0.94.

### 2.4. Data Analysis

The data were analyzed using the SPSS 25.0 software package. Descriptive statistics, including the arithmetic mean, standard deviation, and frequency values, were used to present the statistical representation of the data. The skewness and kurtosis values (+1.5 and −1.5) were considered for normality tests. For values indicating normal distribution, parametric tests were applied: independent *t*-test for pairwise comparisons, ANOVA for multiple comparisons, and Pearson correlation analysis for detecting relationships between variables. Additionally, regression analysis was conducted. To assess the reliability of the questionnaires, the α-Cronbach’s internal consistency coefficient was applied. The sample power was determined using the G*Power indicator.

## 3. Results

This section of the study presents the analysis results and interpretations of the data obtained.

The analysis results (Table 1) indicate that there are statistically significant differences based on gender in the Satisfaction subdimension of the Leisure Boredom Scale (*p* < 0.05, t = 2.218), as well as in the Social Interaction (*p* < 0.05, t = 2.594), Shopping (*p* < 0.05, t = 6.158), and Multimedia Usage (*p* < 0.05, t = 5.144) subdimensions of the Leisure Internet Usage Scale (*p* < 0.05). However, no statistically significant differences were found by gender in the Boredom subdimension of the Leisure Boredom Scale, the Information/Self-improvement subdimension of the Leisure Internet Usage Scale, or the subdimension scores of the Gambling Motives Scale. When examining the scale scores where significant differences were identified, it was noted that women had higher average scores than men in the Satisfaction subdimension of the Leisure Boredom Scale.

In Table 2, the analysis results indicate a significant difference in the Boredom subdimension scores of the Leisure Boredom Scale (*p* < 0.05), while no significant difference was found in the Satisfaction subdimension scores (*p* > 0.05). For the Leisure Internet Usage Scale, statistically significant differences were observed in all subdimensions based on the Leisure Activity Type variable (*p* < 0.05). When examining the Gambling Motives Scale subdimensions, statistically significant differences were noted in the Avoidance and Monetary Gains subdimensions (*p* < 0.05). In contrast, no significant differences were found in the Amusement/Excitement and Socialization subdimensions (*p* > 0.05).

When analyzing the scale subdimension scores with significant differences, it was found that in the Boredom subdimension of the Leisure Boredom Scale, individuals who preferred resting during their leisure time had higher average scores than those who preferred other activities. Regarding the Leisure Internet Usage Scale, those who preferred resting during leisure time had higher average scores in the Social Interaction, Shopping, and Multimedia Usage subdimensions than those engaged in physical or social/cultural activities. However, in the Information/Self-Improvement subdimension of the Leisure Internet Usage Scale, participants engaged in social/cultural activities during their leisure time had higher average scores than those involved in other activities. For the Gambling Motives Scale, individuals participating in physical activities during their leisure time had higher average scores in the Avoidance and Monetary Gains subdimensions than those engaged in other activities.

The analysis results (Table 3) reveal a significant difference in the Boredom subdimension of the Leisure Boredom Scale based on age (*p* < 0.05). In contrast, no difference was found in the Satisfaction subdimension based on age (*p* > 0.05). For the Leisure Internet Usage Scale, statistically significant differences were observed in all subdimensions according to age (*p* < 0.05). Similarly, statistically significant differences were found in all subdimensions of the Gambling Motives Scale based on age (*p* < 0.05). Analyzing the subdimensions where significant differences were observed, it was found that, in the Boredom subdimension of the Leisure Boredom Scale, individuals aged 30 and under had higher average scores compared to those aged 31 and above. For the Leisure Internet Usage Scale, it was found that as participants’ age increased, their average scores decreased in the Social Interaction, Shopping, and Multimedia Usage subdimensions. However, in the Information/Self-Improvement subdimension, individuals aged 31–45 had higher average scores than those aged 30 and under and 46 and above. In all the subdimensions of the Gambling Motives Scale, individuals aged 30 and under had higher average scores than those aged 31 and above.

The correlation analysis in Table 4 shows a statistically significant positive relationship between boredom in leisure time, leisure internet use, and gambling motives (*p* < 0.01).

According to Table 5, the Leisure Boredom and Leisure Internet Usage variables show a significant positive relationship with the dependent variable, Gambling Motives (R = 0.436, R^2^ = 0.190, *p* < 0.05). The standardized regression coefficients reveal that Gambling Motives is most influenced by the Leisure Boredom variable, followed by the Leisure Internet Usage variable. Both independent variables significantly affect Gambling Motives (*p* < 0.05). The high values of the Variance Inflation Factor (VIF) suggest multicollinearity. However, the test results indicate that there is no issue with multicollinearity. A VIF value greater than 5 signifies a multicollinearity problem [32]. Therefore, this study concludes that there is no multicollinearity problem. The Durbin–Watson value tests for autocorrelation, and a value between 0 and 4 is preferred. A Durbin–Watson value between 1.5 and 2.5 indicates no autocorrelation. In this study, the value was found to be 1.690.

## 4. Discussion

This section of the findings from the research conducted to determine adults’ perceptions of boredom during leisure time, their internet usage patterns in leisure, and the reasons behind gambling, together with an analysis of the predictive effects by examining the relationships among these variables, is discussed alongside the literature.

The first finding of the study highlights gender differences in participants’ perceptions of leisure, internet use, and gambling behaviors. In the Satisfaction subdimension of leisure boredom perception, women scored higher than men. This aligns with recent literature suggesting that women are more likely to seek emotional satisfaction from their leisure activities than men. Studies have shown that emotional and relational factors often influence women’s leisure choices, while men prioritize activities that provide relaxation or physical engagement challenges [33,34]. Furthermore, research shows that women report greater levels of emotional well-being and satisfaction in leisure activities involving social interaction and community [35,36]. Additionally, it was observed that women preferred digital platforms such as social interaction, shopping, and multimedia use more than men. This aligns with findings indicating that women are more likely to use the internet for social connections and entertainment [37,38,39]. Recent studies suggest that women are more likely to engage with digital content that fosters emotional expression and social bonding, highlighting their preference for online social interactions over those of men [40,41]. However, no significant gender differences were found in the Information/Self-improvement subdimension of leisure boredom and internet use. Recent studies have further supported this, indicating that both men and women engage in internet-based self-improvement and information-seeking behaviors similarly, despite differences in overall internet usage patterns [41,42]. Regarding gambling motivations, both genders were observed to share similar motivations for gambling, which aligns with studies showing that, although men typically gamble more frequently, their motivations are indeed quite similar to those of women [43,44]. Overall, these findings contribute to understanding the role of gender in leisure activities.

Another finding from the study examines participants’ leisure boredom, internet use, and gambling motivations based on their preferred leisure activities. In the boredom subdimension of the Leisure Boredom Scale, individuals who preferred resting during leisure scored higher (mean = 2.73) than those engaged in other activities. This finding aligns with recent literature suggesting that rest-related activities may evoke monotony more strongly, as they do not provide the stimulation and engagement that active or social leisure activities offer [45,46]. Moreover, research indicates that individuals who engage in less stimulating leisure activities are at a higher risk of experiencing negative emotional states such as boredom and dissatisfaction [47]. Furthermore, in the Leisure Internet Usage Scale, individuals who preferred to rest had higher average scores in Social Interaction, Shopping, and Multimedia Usage subdimensions. This observation aligns with findings indicating that the internet is used more frequently for entertainment and social connection by individuals who engage less in physical or social/cultural activities [25]. However, in the Information/Self-improvement subdimension, participants who engaged in social and cultural activities scored higher, indicating that these activities more effectively fulfill individuals’ needs for information acquisition and personal development. Regarding the Gambling Motives Scale, individuals participating in physical activities scored higher in the Avoidance and Monetary Gains subdimensions. This suggests that physical activities may trigger stress relief and avoidance behaviors while increasing the desire for monetary rewards through risky behaviors. One of them is gambling, a finding that aligns with previous studies [46,48,49]. Furthermore, research indicates that physical activity can heighten the psychological need for excitement, potentially contributing to increased gambling and behavior [25]. These findings provide valuable insights into how individuals’ leisure activity choices relate to physical, psychological, and social factors.

Another finding from the study revolves around participants’ leisure boredom, internet usage, and gambling motivations based on age. In the Boredom subdimension of the Leisure Boredom Scale, individuals aged 30 and under scored higher than those aged 31 and above. This result aligns with previous studies that suggest younger individuals may experience a greater sense of monotony and boredom [5]. In the Leisure Internet Usage Scale, it was observed that the average scores of participants in the Social Interaction, Shopping, and Multimedia Usage subdimensions decreased as age increased. This observation aligns with the literature indicating that internet usage patterns may differ by age, with older individuals showing less interest in digital content than younger individuals [25]. In the Information/Self-improvement subdimension, individuals aged 31–45 scored higher than those aged 30 and younger and those aged 46 and older. This suggests that middle-aged individuals may engage more in internet usage related to personal development and education. Regarding the Gambling Motives Scale, individuals aged 30 and younger scored higher in all subdimensions than those aged 31 and older. This finding aligns with recent research indicating that younger individuals are more likely to engage in risky behaviors, such as gambling, viewing them as excitement-seeking activities. Studies have shown that younger adults tend to exhibit higher levels of sensation-seeking and risk-taking behaviors, which may contribute to their greater engagement in gambling [50,51,52]. Furthermore, research indicates that the desire for novelty and thrill-seeking significantly contributes to increased gambling motivation and behavior among younger individuals [53]. Overall, the findings from the study highlight that age is a crucial factor influencing how individuals engage in leisure activities, internet usage, and risky behaviors such as gambling.

When examining the findings in Table 4, a statistically significant and robust positive relationship was identified between Leisure Boredom, Leisure Internet Usage, and Gambling Motives (*p* < 0.01). This result indicates that as individuals experience higher levels of boredom during their leisure time, their motivations for internet usage and gambling behaviors also increase. This finding supports recent studies suggesting that boredom can trigger both internet usage and gambling behavior. Research has shown that individuals experiencing boredom are more likely to engage in maladaptive behaviors, including excessive internet use and gambling, to escape negative emotions [38,54]. Furthermore, studies indicate that boredom may enhance the allure of risky behaviors such as gambling, as individuals pursue stimulation or excitement to relieve their feelings of monotony [49,50]. These activities offer the stimulation and emotional engagement that individuals experiencing higher levels of boredom may seek to alleviate their negative emotional states [55,56]. Moreover, this finding suggests that leisure time, beyond relaxation or entertainment, can also function as a mechanism for managing psychological discomfort, especially feelings of boredom [5]. The significant positive relationship noted in this study highlights the necessity of addressing the negative aspects of leisure time, particularly boredom, to prevent the emergence of unhealthy behaviors such as excessive internet use and gambling. In this context, boredom experienced during individuals’ leisure time emerges not merely as a transient emotional state but as a significant psychological factor that may predispose individuals to engage in risky and maladaptive behaviors. This underscores the direct impact of how individuals structure their leisure time on the overall quality of life. Therefore, prioritizing developing and implementing effective leisure time management strategies is critical for enhancing well-being.

According to the findings in Table 5, the Leisure Boredom and Leisure Internet Usage variables exhibit a positive and significant relationship with Gambling Motives. The regression analysis indicates that Gambling Motives are more strongly influenced by the Leisure Boredom variable, followed by the Leisure Internet Usage variable. This finding aligns with previous studies in the literature. Specifically, research suggesting that boredom can trigger risky behaviors, such as gambling, supports the results of this study [43]. Furthermore, studies have emphasized that the experience of boredom can drive individuals to spend more time on digital platforms, potentially fostering addictive behaviors like gambling [25,53]. These findings reveal that boredom experienced during leisure time and increased engagement with digital platforms may predispose individuals to risky behaviors such as gambling. This suggests that how leisure time is structured is not merely a matter of personal preference but a critical factor influencing psychological well-being and overall quality of life. In this regard, it is argued that policies and interventions to enhance quality of life should incorporate strategies that promote more conscious and meaningful engagement with leisure activities.

The study’s findings indicate that internet usage is also related to social and cultural subdimensions, particularly Social Interaction and Shopping. Using the internet in this way leads individuals to spend more time in digital environments, making risky behaviors such as gambling more accessible and anonymous [23,51,57]. Furthermore, this study supports the idea that addictive behaviors, like gambling, are not limited to individual experiences; they are also influenced by environmental and digital factors, which provide continuous access and opportunities for engagement. In this context, it can be asserted that the unlimited access provided by digital environments significantly influences individuals’ leisure behaviors, which may adversely affect their quality of life over time. Notably, digital activities that initially seem relatively benign, such as social interaction and shopping, have the potential to lead individuals toward riskier behaviors when used uncontrollably. Therefore, to preserve and enhance quality of life, it is imperative to raise awareness about digital leisure activities, develop strategies that prioritize digital balance, and strengthen leisure time management skills.

### 4.1. Limitations and Future Directions of the Study

Among the study’s limitations, we mention the cross-sectional design, the absence of an intervention program, and the exclusion of socio-economic status and education level information in the analysis of demographic variables. Another limitation concerns the instruments used for data collection—the three self-assessment questionnaires—which may introduce distortions in the accuracy of the data, particularly relating to the reporting of stigmatizing behaviors such as internet usage habits or participation in gambling. The study did not consider other factors that could influence the observed relationships, including stress levels, sleep disorders, depression, or anxiety—variables that may significantly relate to both internet usage habits, digital addiction, and gambling behaviors during the night. Additionally, the predominance of male participants may limit the generalizability of the findings, particularly in cases where no gender-based comparisons are made. It is recommended that future studies employ samples with a more balanced gender distribution.

Future studies should explore the relationship between leisure boredom, different age groups, and socio-economic factors in more detail, especially in light of the changing leisure habits during the post-pandemic period. Additionally, long-term longitudinal studies should be initiated to thoroughly investigate how the negative use of leisure time affects individuals’ psychological well-being and overall quality of life. Another research avenue focusing on digital addiction may involve assessing its reduction through well-defined and targeted awareness programs designed to help individuals balance their internet usage and raise awareness regarding excessive use of social networks and online gaming platforms.

### 4.2. The Practical Implications of the Study 

The study’s practical implications involve the prevention and management of risk behaviors associated with underutilized free time, providing concrete data for specialists in the field. From a psychological perspective, the finding that boredom can significantly influence the correlation between internet usage habits, digital addiction, and nocturnal gambling behaviors highlights the need to develop psychological intervention programs aimed at individuals. The recorded data can serve as a valuable practical basis for specialists in understanding the factors contributing to behavioral addictions and facilitating therapeutic interventions. The findings regarding the prevalence of gambling behaviors at night can support initiatives to regulate access to these platforms. Public policies could aim to restrict access during specific time intervals, introduce automatic alert systems for users who spend excessive time on these platforms, or promote self-exclusion mechanisms.

## 5. Conclusions

In conclusion, the findings of this study highlight the significant role of leisure boredom as a psychological factor that correlates with increased tendencies toward internet use and gambling activities. The positive association identified between leisure boredom, internet usage habits, and high-risk behaviors emphasizes the necessity of effectively managing leisure time to enhance psychological well-being and promote healthier behavioral patterns. Furthermore, the direct relationship established between internet usage and gambling motivations indicates that digital platforms play a crucial role in facilitating gambling behaviors, primarily due to their inherent anonymity and ease of access. Age-related differences noted in the study reveal that younger individuals experience leisure boredom more intensely, subsequently influencing their internet usage patterns and gambling behaviors. These insights are essential for guiding the creation of targeted interventions focused on at-risk groups, especially young adults, to reduce the potential negative effects of these behaviors.

## Figures and Tables

**Table 1:** Leisure Boredom, Leisure Internet Usage, and Gambling Motives Scales *t*-test result according to gender variable.

Scale	Factor	Gender	n	X	SD	t	*p*
Leisure Boredom	Boredom	Female	96	2.42	0.815	0.274	0.78
		Male	214	2.40	0.767		
	Satisfaction	Female	96	3.66	0.617	2.218	0.03 *
		Male	214	3.48	0.716		
Leisure Internet Usage	Social İnteraction	Female	96	3.58	0.827	2.594	0.01 *
		Male	214	3.31	0.864		
	Shopping	Female	96	3.19	0.896	6.158	0.00 *
		Male	214	2.50	0.922		
	Multimedia Usage	Female	96	3.44	0.831	5.144	0.00 *
		Male	214	2.89	0.886		
	İnformation/Self-improvement	Female	96	3.74	0.708	−1.671	0.09
		Male	214	3.88	0.687		
Gambling Motives	Amusement/Excitement	Female	96	2.16	0.919	−0.569	0.56
		Male	214	2.23	0.948		
	Avoidance	Female	96	1.98	0.869	−0.129	0.89
		Male	214	1.99	0.833		
	Monetary Gains	Female	96	2.06	0.939	0.635	0.52
		Male	214	1.99	0.856		
	Socialization	Female	96	2.10	0.956	−1.095	0.27
		Male	214	2.23	0.925		

* *p* < 0.05; n—number of subjects; X—arithmetic mean; SD—standard deviation; t—*t*-test; *p*—statistical significance values.

**Table 2:** Leisure Boredom, Leisure Internet Usage, and Gambling Motives Scales ANOVA result according to the Leisure Activity Type variable.

Scale	Factor	Leisure Type	N	Mean	SD	F	*p*
Leisure Boredom	Boredom	Rest	99	2.73	0.875	14.118	0.00 *
		Physical Activity	83	2.33	0.705		
		Social/Cultural Activity	128	2.21	0.669		
		Rest	99	3.40	0.614	2.828	0.06
	Satisfaction	Physical Activity	83	3.61	0.728		
		Social/Cultural Activity	128	3.60	0.712		
Leisure Internet Usage	Social İnteraction	Rest	99	3.64	0.827		
		Physical Activity	83	3.19	0.852	6.892	0.00 *
		Social/Cultural Activity	128	3.33	0.853		
	Shopping	Rest	99	3.04	0.947		
		Physical Activity	83	2.71	0.926	10.530	0.00*
		Social/Cultural Activity	128	2.46	0.940		
	Multimedia Usage	Rest	99	3.37	0.895	10.279	0.00 *
		Physical Activity	83	3.04	0.943		
		Social/Cultural Activity	128	2.84	0.819		
	İnformation/Self-improvement	Rest	99	3.78	0.726	4.182	0.01 *
		Physical Activity	83	3.70	0.707		
		Social/Cultural Activity	128	3.96	0.645		
Gambling Motives	Amusement/Excitement	Rest	99	2.22	1.032	1.568	0.21
		Physical Activity	83	2.34	0.975		
		Social/Cultural Activity	128	2.11	0.828		
	Avoidance	Rest	99	2.14	0.978	5.243	0.00 *
		Physical Activity	83	2.07	0.866		
		Social/Cultural Activity	128	1.81	0.671		
	Monetary Gains	Rest	99	2.08	0.969	4.956	0.00 *
		Physical Activity	83	2.21	0.945		
		Social/Cultural Activity	128	1.84	0.729		
	Socialization	Rest	99	2.18	1.027	2.043	0.13
		Physical Activity	83	2.36	0.959		
		Social/Cultural Activity	128	2.09	0.832		

* *p* < 0.05; N—number of subjects; SD—standard deviation; F—test value; *p*—probability level.

**Table 3:** Leisure Boredom, Leisure Internet Usage, and Gambling Motives Scales ANOVA result according to the Age variable.

Scale	Factor	Age	N	Mean	SD	F	*p*
Leisure Boredom	Boredom	30 years old and under	108	2.59	0.854	5.008	0.00 *
		Between 31 and 45 years old	71	2.37	0.885		
		46 years old and above	130	2.27	0.619		
		30 years old and under	108	3.65	0.604	3.070	0.04
	Satisfaction	Between 31 and45 years old	71	3.59	0.760		
		46 years old and above	130	3.44	0.685		
Leisure Internet Usage	Social İnteraction	30 years old and under	108	3.81	0.696		
		Between 31 and 45 years old	71	3.32	0.838	24.181	0.00 *
		46 years old and above	130	3.09	0.854		
	Shopping	30 years old and under	108	3.17	0.866		
		Between 31 and 45 years old	71	2.90	0.933	35.419	0.00 *
		46 years old and above	130	2.24	0.840		
	Multimedia Usage	30 years old and under	108	3.68	0.717	65.917	0.00 *
		Between 31 and 45 years old	71	3.08	0.796		
		46 years old and above	130	2.55	0.770		
	İnformation/Self-improvement	30 years old and under	108	3.82	0.689	2.574	0.07
		Between 31 and 45 years old	71	3.99	0.620		
		46 years old and above	130	2.95	0.726		
Gambling Motives	Amusement/ Excitement	30 years old and under	108	2.53	0.985	10.197	0.00 *
		Between 31 and 45 years old	71	2.00	1.000		
		46 years old and above	130	2.05	0.793		
	Avoidance	30 years old and under	108	2.30	0.975	12.158	000 *
		Between 31 and 45 years old	71	1.82	0.841		
		46 years old and above	130	1.82	0.636		
	Monetary Gains	30 years old and under	108	2.42	0.964	19.893	0.00 *
		Between 31 and 45 years old	71	1.83	0.863		
		46 years old and above	130	1.78	0.688		
	Socialization	30 years old and under	108	2.45	1.018	6.732	0.00 *
		Between 31 and 45 years old	71	2.00	0.949		
		46 years old and above	130	2.08	0.809		

* *p* < 0.05; N—number of subjects; SD—standard deviation; F—test value; *p*—probability level.

**Table 4:** Results of correlation analysis between variables.

Variables	Leisure Boredom		Leisure Internet Usage	Gambling Motives
Leisure Boredom	r	1	0.335 **	0.379 **
	*p*		0.001	0.001
Leisure Internet Usage	r	0.335 **	1	0.330 **
	*p*	0.001		0.001
Gambling Motives	r	0.379 **	0.330 **	1
	*p*	0.001	0.001	

** *p* < 0.01.

**Table 5:** Leisure Boredom and Leisure Internet Usage as a predictor of Gambling Motives.

Model	B	SD	β	T	*p*	Binary	Partial	Tolerance	VIF	Durbin–Watson
Constant	−0.617	0.326		−1.893	0.059					1.690
Leisure Boredom	0.577	0.104	0.303	5.550	0.001	0.379	0.302	0.888	1.127	
Leisure Internet Usage	0.311	0.074	0.229	4.201	0.001	0.330	0.233	0.888	1.127	
R = 0.436, R^2^ = 0.190 F = 36.101, *p* < 0.001										

SD—standard deviation; VIF—Variance Inflation Factor.

## Data Availability

The datasets generated and analyzed during the current study are not publicly available for privacy reasons.

## References

[1] Iso-Ahola S.E. (1980). The Social Psychology of Leisure and Recreation.

[2] Coleman D., Iso-Ahola S.E. (1993). Leisure and Health: The Role of Social Support and Self-Determination. J. Leis. Res..

[3] Mannell R.C., Kleiber D.A. (1997). A Social Psychology of Leisure.

[4] Rupp L., Vodanovich S.J. (1997). The role of boredom proneness in self-reported anger and aggression. J. Soc. Behav. Personal..

[5] Bench S.W., Lench H.C. (2013). On the function of boredom. Behav. Sci..

[6] Robinson J.P., Godbey G. (1997). Time for Life: The Surprising Ways Americans Use Their Time.

[7] Satılmış S.E., Cengız R., Güngörmüş H.A. (2023). The perception of leisure boredom and internet addiction in university students during social isolation. J. Depend..

[8] Ayhan C., Kaya H.B., Yalçın I., Karakaş G. (2023). The mediating effect of social media addiction on the relationship between leisure boredom and loneliness. J. Hum. Sci..

[9] Cannito L., Ceccato I., Annunzi E., Bortolotti A., D’intino E., Palumbo R., D’addario C., Di Domenico A., Palumbo R. (2023). Bored with boredom? Trait boredom predicts internet addiction through the mediating role of attentional bias toward social networks. Front. Hum. Neurosci..

[10] Young K.S. (1998). Internet addiction: The emergence of a new clinical disorder. CyberPsychology Behav..

[11] Griffiths M.D. (2005). A “components” model of addiction within a biopsychosocial framework. J. Subst. Use.

[12] Kelly J.R. (1996). Leisure: Perspectives on the limits of choice. J. Leis. Res..

[13] Kuss D.J., Griffiths M.D. (2017). Social networking sites and addiction: Ten lessons learned. Int. J. Environ. Res. Public Health.

[14] Tam K.Y.Y., Inzlicht M. (2024). People are increasingly bored in our digital age. Commun. Psychol..

[15] Camerini A.-L., Morlino S., Marciano L. (2023). Boredom and digital media use: A systematic review and meta-analysis. Comput. Hum. Behav. Rep..

[16] Bessière K., Seay F., Kiesler S. (2010). The ideal self and the role of online communities. Comput. Hum. Behavior..

[17] Sahin M., Lok S. (2018). Relationship between Physical Activity Levels and Internet Addiction of Adults. J. Depress. Anxiety.

[18] Chen B.C., Chen M.Y., Wu Y.F., Wu Y.T. (2022). The Relationship of Social Media Addiction with Internet Use and Perceived Health: The Moderating Effects of Regular Exercise Intervention. Front. Public Health.

[19] Chen X.-L., Li J., Sun S.-N., Zhao Q.-Q., Lin S.-R., Wang L.-J., Yang Z.-Q., Ni S.-H., Lu L. (2024). Association Between Daily Internet Use and Intrinsic Capacity Among Middle-Aged and Older Adults in China: Large Prospective Cohort Study. J. Med. Internet Res..

[20] Griffiths M.D. (2004). Gambling Addiction: A Handbook for Therapists.

[21] Goldstein A.L., Vilhena-Churchill N., Stewart S.H., Hoaken P.N., Flett G.L. (2016). Mood, motives, and money: An examination of factors that differentiate online and non-online young adult gamblers. J. Behav. Addict..

[22] Wirkus T., Czernecka R., Bühringer G., Kräplin A. (2024). Individual risk factors and prediction of gambling disorder in online sports bettors—The longitudinal RIGAB study. Front. Psychiatry.

[23] Griffiths M.D., Auer M. (2013). Online gambling. The Wiley-Blackwell Handbook of the Psychology of Gambling.

[24] Blaszczynski A., Nower L. (2002). A pathways model of problem and pathological gambling. Addiction.

[25] Brooks G.A., Clark L. (2022). Gambling along the schizotypal spectrum: The associations between schizotypal personality, gambling-related cognitions, luck, and problem gambling. J. Behav. Addict..

[26] Gainsbury S.M., Blaszczynski A. (2015). Online Gambling Addiction: The Relationship Between Internet Gambling and Disordered Gambling. Curr. Addict. Rep..

[27] Iso-Ahola S.E., Weissinger E. (1990). Perceptions of boredom in leisure: Conceptualization, reliability, and validity of the leisure boredom scale. J. Leis. Res..

[28] Kara F.M., Gürbüz B., Öncü E. (2014). Leisure boredom scale: The factor structure and the demographic differences. Turk. J. Sport Exerc..

[29] Şimşek K.Y., Çevik H. (2023). The development and validation of the Leisure Internet Usage Scale (LIUS). Leis. Stud..

[30] Lee H.P., Chae P.K., Lee H.S., Kim Y.K. (2007). The five-factor gambling motivation model. Psychiatry Res..

[31] Arcan K., Karancı A.N. (2014). Kumar Oynama Nedenleri Ölçeğinin uyarlama, geçerlilik ve güvenilirlik çalışması [Adaptation, validity, and reliability study of the Five-Factor Gambling Motives Scale]. Anadolu Psikiyatr. Derg..

[32] Montgomery D.C., Peck E.A., Vining G.G. (2012). Introduction to Linear Regression Analysis.

[33] Tabachnick B.G., Fidell L.S. (2013). Using Multivariate Statistics.

[34] Hassing L.B. (2020). Gender Differences in the Association Between Leisure Activity in Adulthood and Cognitive Function in Old Age: A Prospective Longitudinal Population-Based Study. J. Gerontol. Ser. B Psychol. Sci. Soc. Sci..

[35] Ahn B.-W., Song W.-I. (2021). A Study of Differences in Leisure Satisfaction of Leisure Activity Patterns for South Korean Adults. Int. J. Environ. Res. Public Health.

[36] Fu A., Yang T., Wen P., Yin Z. (2025). Impact of different leisure participation patterns on stress coping and authentic happiness in married women. Leis. Stud..

[37] Yerkes M.A., Roeters A., Baxter J. (2018). Gender differences in the quality of leisure: A cross-national comparison. Community Work. Fam..

[38] Twenge J.M., Martin G.N. (2020). Gender differences in associations between digital media use and psychological well-being: Evidence from three large datasets. J. Adolesc..

[39] Jeong H., Yim H.W., Lee S., Lee H.K., Potenza M.N., Jo S., Son H.J., Kim G. (2020). Low self-control and aggression exert serial mediation between inattention/hyperactivity problems and severity of internet gaming disorder features longitudinally among adolescents. J. Behav. Addict..

[40] Lamberti G., López-Sintas J., Lopez Belbeze P. (2023). The impact of internet use on leisure: Gender and age heterogeneity in young people. J. Leis. Res..

[41] Mannell R.C. (2007). Leisure, Health and Well-Being. World Leis. J..

[42] Lee Y.K. (2022). Gender differences in leisure: From the relationship between leisure type-time and time use satisfaction in Korea. World Leis. J..

[43] Li Z., Lu F. (2024). The power of Internet: From the perspective of women’s bargaining power. Humanit. Soc. Sci. Commun..

[44] Wong G., Zane N., Saw A., Chan A.K. (2013). Examining gender differences for gambling engagement and gambling problems among emerging adults. J. Gambl. Stud..

[45] Zhang K., Clark L. (2020). Loss-chasing in gambling behaviour: Neurocognitive and behavioural economic perspectives. Curr. Opin. Behav. Sci..

[46] Sezer K.S., Aki E. (2024). “It Is as if I Gave a Gift to Myself”: A Qualitative Phenomenological Study on Working Adults’ Leisure Meaning, Experiences, and Participation. Behav. Sci..

[47] Nowotarski A., Rothen S., Kasina F., Zullino D., Thorens G. (2024). Physical Activity as a Predictor of Internet Gaming Disorder in a Swiss Male Cohort (C-SURF): L’activité physique comme prédicteur des troubles liés aux jeux vidéo en ligne dans une cohorte de jeunes hommes suisses (C-SURF). Can. J. Psychiatry.

[48] Alecu S., Ionescu-Bondoc D. (2024). Proprioception testing in junior athletes training using the “gyko” sensor. Bull. Transilv. Univ. Braşov. Ser. IX: Sci. Hum. Kinet..

[49] Miller H., Brown A., Taylor S. (2020). Physical activity and gambling: The role of stress and risk-taking behavior. J. Gambl. Stud..

[50] Koomson I., Churchill S.A., Munyanyi M.E. (2022). Gambling and Financial Stress. Soc. Indic. Res..

[51] Williams R., O’Connor M. (2021). Digital environments, and addictive behaviors: How virtual platforms influence gambling addiction. Cyberpsychology Behav. Soc. Netw..

[52] An J., Payne L.L., Lee M., Janke M.C. (2023). Understanding Boredom and Leisure in Later Life: A Systematic Review. Innov. Aging.

[53] Hing N., Rockloff M., Russell A.M.T., Browne M., Newall P., Greer N., King D.L., Thorne H. (2020). Loot box purchasing is linked to problem gambling in adolescents when controlling for monetary gambling participation. J. Behav. Addict..

[54] Zhang D., Zhou S., Zheng H., Guo L., Zhai J., Liu Z., Du Z., Dong P., Zhao M., Du J. (2023). How general functioning of family affects gambling-related beliefs: The mediating role of communication and the moderating role of impulsivity trait. Front. Psychiatry.

[55] Yan Z., Yang Z., Xu X., Zhou C., Sang Q. (2025). Problematic Online Video Watching, Boredom Proneness and Loneliness Among First-Year Chinese Undergraduates: A Two-Wave Longitudinal Study. Psychol. Res. Behav. Manag..

[56] Rogier G., Velotti P. (2018). Conceptualizing gambling disorder with the process model of emotion regulation. J. Behav. Addict..

[57] Cho T.H., Nah Y., Park S.H., Han S. (2022). Prefrontal cortical activation in Internet Gaming Disorder Scale high scorers during actual real-time internet gaming: A preliminary study using fNIRS. J. Behav. Addict..

